# The Secondary Harms of Parental Substance Use on Children’s Educational Outcomes: A Review

**DOI:** 10.1007/s40653-021-00433-2

**Published:** 2022-01-14

**Authors:** Emily Lowthian

**Affiliations:** 1 DECIPHer, School of Social Sciences, Cardiff University, 1–3 Museum Place, Cardiff, CF10 3BD Wales, UK; 2 Population Data Science, Swansea University Medical School, Singleton Park, Swansea, SA2 8PP Wales, UK

**Keywords:** Alcohol, Drugs, Parental substance use, Education, Review

## Abstract

Parental substance use, that is alcohol and illicit drugs, can have a deleterious impact on child health and wellbeing. An area that can be affected by parental substance use is the educational outcomes of children. Current reviews of the literature in the field of parental substance use and children's educational outcomes have only identified a small number of studies, and most focus on children's educational attainment. To grasp the available literature, the method from Arksey and O’Malley (2005) was used to identify literature. Studies were included if they were empirical, after 1950, and focused on children’s school or educational outcomes. From this, 51 empirical studies were identified which examined the relationship between parental alcohol and illicit drug use on children’s educational outcomes. Five main themes emerged which included attainment, behavior and adjustment, attendance, school enjoyment and satisfaction, academic self-concept, along with other miscellaneous outcomes. This paper highlights the main findings of the studies, the gaps in the current literature, and the challenges presented. Recommendations are made for further research and interventions in the areas of parental substance use and child educational outcomes specifically, but also for broader areas of adversity and child wellbeing.

## Background

Parental alcohol and illicit drug use can have deleterious and enduring impacts on their children’s health and wellbeing (Kuppens et al., 2020; McGovern et al., 2018; Park & Schepp, 2015; Velleman & Templeton, 2016). Research has found that children who grow up in environments where their parents use substances are at higher risk for externalizing symptoms, such as hyperactivity (Finan et al., 2015; Hussong et al., 2010) and also internalizing symptoms, such as depression or anxiety (Lee & Cranford, 2008; Pisinger et al., 2016). These children can also be at greater risk for injury or infectious diseases (Crandall et al., 2006; Raitasalo et al., 2015). Furthermore, a large body of evidence highlights that parental substance use is associated with their own children’s substance use in adolescence (Chassin et al., 1993, 1996, 1999; Hussong et al., 2012).

While reviews of the literature on parental substance use have often included many health and wellbeing outcomes in their search strategy and subsequent findings, they continue to report a low number of studies regarding educational outcomes (Kuppens et al., 2020; McGovern et al., 2018; Park & Schepp, 2015; Velleman & Templeton, 2016). For instance, in Kuppens et al. (2020) only one study was identified that examined the longitudinal relationship between parental alcohol use and children’s academic achievement (McGrath et al., 1999). Likewise, McGovern et al. (2018) found three studies in their rapid review, and stated that there was a lack of research which considered children's educational outcomes following non-dependent parental substance use. Other reviews of evidence also cite a lower number of studies regarding children's educational outcomes, and perhaps this explains why education outcomes are not discussed at length compared to other health and wellbeing outcomes (Park & Schepp, 2015; Velleman & Templeton, 2016).

The lower attention given to educational outcomes in other reviews of literature is a concern when educational outcomes permit access to further and higher education, which have implications for later employment opportunities. Indeed, Howieson and Iannelli (2008) found that those who had lower educational attainment had poorer labor market outcomes by the time they were 22 to 23 years old. Given employment opportunities influence socioeconomic status, which is related to later health and wellbeing (Marmot, 2005) and disability free years (Melzer, 2000) a negative impact could have implications for life opportunities and satisfaction.

This is further warranted when problem substance use is concernedly prevalent. Recent estimates suggest that 3.7% of children have a parent known to alcohol and drug services, 15—24% have parents who used illicit drugs in the last year, and 14—37% have a parent who has an alcohol dependency (Galligan & Comiskey, 2019); these estimates are higher than older estimates presented by Manning et al. (2009). As a result, a large proportion of children could be at risk for lower educational outcomes and denied subsequent life opportunities. Therefore, as previous reviews in this area have found a small number of studies, a wider search strategy will be used to capture the extent of research exploring the relationship between parental substance use, that is alcohol or illicit drugs, and children’s educational outcomes.

## Methods

The review aimed to map out and synthesize the current research, while identifying the gaps in existing literature to draw conclusions from overall activity (Arksey & O’Malley, 2005). The review was conducted using the guidance from Arksey and O’Malley, (2005) as the research question was broad and not well defined, and there was no quest to find answers from a narrow range of quality assessed studies. The guidance provides five steps: research question; identification; study selection; charting data; collating and summarizing the results.

### Research Question

As in the guidelines, the research question remained broad—What is the relationship between parental substance use and children’s educational outcomes?

### Identifying Relevant Studies

The search for literature started from late 2017 to mid-2018 using an array of sources. First, electronic databases were searched to identify literature using keywords such as ‘parental substance use’, ‘maternal alcohol’, ‘children of alcoholics’, ‘school attainment’, ‘school outcomes’, ‘educational achievement’, ‘academic attainment’ etc. Electronic searches were largely conducted on Google Scholar as grey literature, published studies, and doctoral theses could be identified simultaneously. Second, a hand searching of reports from key organizations (e.g., NSPCC) was conducted to identify literature. Third, the references of each source included were traced to identify further literature. Fourth, the citations were traced on all sources that were included. Lastly, existing networks, relevant organizations and conferences were used to identify any further literature. Once the main search had been conducted, a citation alert on highly cited papers in the area (e.g., Berg et al., 2016; Torvik et al., 2011) was created on Google Scholar to ensure the review was updated, and references and citations were traced as before. The inclusion of any research which was published after 2018, is a result of communication with existing networks or the citation alert.

### Study Selection

The title was reviewed initially to determine the relevance of the publication, if relevant the abstract would be reviewed, and then the full-text. The inclusion criteria required the evidence to be empirical, which focused on both parental substance use *and* children’s educational (or school) outcomes; measures of IQ and cognitive functioning often appeared but were excluded as these coincided less with the educational system. Journal articles, doctoral theses, and other grey literature was included as it was peer reviewed; reviews of evidence were used only to identify literature via references or citations. The exclusion criteria included undergraduate or master’s dissertations, articles not in English, and research that was conducted before 1950. Articles that were not available via Cardiff University were requested via inter-library loans. Key literature was identified until a saturation point was reached where no new literature was identified (Arksey & O’Malley, 2005).

### Charting of Data

The information of each empirical study was recorded in Microsoft Excel, as in Arksey and O’Malley (2005). The details included author; year of publication; study location; study population; measurement of substance use; study aims; methods; outcome measures, and key findings. This is approach is described more similar to a narrative review, as it uses a ‘describe-analytical’ method (Arksey & O’Malley, 2005) and was used to collate the findings and identify themes.

### Collating and Summarizing Results

The number of studies, their methods, location, and age could be reported in a descriptive sense. The findings of each study was recorded and collated into areas of subject focus, such as attainment, so useful comparisons could be made across sources and dominant findings could be identified. Through this, the research gaps were also identified and are later discussed.

## Results

The review identified 51 empirical studies and eight reviews (Kuppens et al., 2020; McGovern et al., 2018; Park & Schepp, 2015; Smith, 1993; Velleman & Templeton, 2007, 2016; West & Prinz, 1987; Wilens, 1994). Note, reviews were only used to identify literature and do not feature in the main body of the findings. Table 1 summarizes information on each study, including the population and research focus. Most studies were conducted in the United States (26), Canada (5), Sweden (3), India (3), Denmark (3), and Spain (3). One study was found in each country of Russia, Greece, Slovenia, Brazil, Republic of Ireland, Australia, New Zealand, Finland and Wales, UK; some studies included two countries in their population. Under a third of the studies were published since 2000 (19), 15 in the 1990s, 8 in the 1980s, 6 in the 1970s, and 3 in 1960s. Most of the literature explored parental alcohol use (41), whereas five studies examined illicit drug use, and poly-use. The literature was predominantly quantitative (48) compared to qualitative (2) or mixed-method (1). The findings are summarized in terms of the educational outcome areas of attainment, behavior and adjustment, attendance, academic self-concept, school enjoyment and satisfaction and other miscellaneous outcomes.

**Table 1:** Overview of studies included in review in terms of design, sample and research focus

Author	Design	Study population	Research focus
Evans et al., 2020	Quantitative, longitudinal	*N* = 107,479 and *N* = 43,648 children in Wales from routine data	Explores the effect of adverse childhood experiences on child academic attainment
Raitasalo et al., 2020	Quantitative, longitudinal	*N* = 64,696 and *N* = 64,138 children born in Finland and Denmark in 1991 from routine data	Compares educational attainment among those with parents with alcohol problems and those without
Mangiavacchi & Piccoli, 2018	Quantitative, longitudinal cohort	*N* = 1,740 individuals taken from rounds of a 1994—2014 Russian longitudinal study	Examines whether parental alcohol consumption during childhood can affect long-term educational achievement
Carbonneau et al., 2017	Quantitative, longitudinal	*N* = 653 urban, low socioeconomic status families from a Canadian longitudinal study	Examines school adjustment and substance use when children reside with a father who has alcoholism
Berg et al., 2016	Quantitative, longitudinal cohort	*N* = 740,618 individuals born in Sweden in 1990—1996 from routine data	Studies the relationship between parental alcohol-related disorders and children's school performance
Gifford et al., 2015	Quantitative, longitudinal	*N* = 76,119 children in the conviction sample, and *N* = 842,767 children in the non-conviction sample from routine data	Examines the relationship between parental alcohol or drug use on children's school performance and the impact of drug court treatment interventions
Jennison, 2014	Quantitative, longitudinal	*N* = 4,648 children drawn from a longitudinal study in the US	Studies the impact of parental alcohol misuse and the family environment on young adults school behavior
Pinto & Kulkarni, 2012	Quantitative, comparative	*N* = 107 families in study group and control group	Compares school drop-out among children of alcohol dependent males and matched controls
Serec et al., 2012	Quantitative, comparative	*N* = 57 children of alcoholics and *N* = 87 controls	Compares children of alcoholics school performance with controls
Torvik et al., 2011	Quantitative, cross-sectional	*N* = 8,934 children from a health survey	Investigates the relationship between parental alcohol use and school adjustment
Brook et al., 2010	Quantitative, cross-sectional community sample	*N* = 209 mothers and *N* = 209 children from urban African American and Puerto Rican families who attended schools in East Harlem, New York	Examines pathways to academic achievement via substance use, mother–child relationship, and personality attributes
Jeffreys et al., 2009	Quantitative, cross-sectional	*N* = 467 children entered care system in 2006, *N* = 50 children with recorded parental substance use, and *N* = 50 with none recorded	Compares family circumstances and outcomes for children who entered care from families where substance use was present vs. not present
Díaz et al., 2008	Quantitative, comparative	*N* = 371 children of alcoholics and *N* = 147 controls	Examines school performance across children of alcoholics and matched controls
Zanoti-Jeronymo & Carvalho, 2005	Quantitative, comparative	*N* = 20 children of alcoholics and *N* = 20 controls	Explores whether children of alcoholics are at risk for lower academic and behavioral performance
Casas-Gil & Navarro-Guzman, 2002	Quantitative, comparative	*N* = 108 children of alcoholics and *N* = 118 controls	Investigates the school performance of children of alcoholics and controls
Hogan & Higgins, 2001	Qualitative, multi-level analysis	*N* = 100 parents, *N* = 2 professionals in 26 schools, *N* = 24 children with drug-using parents, *N* = 40 children with non-drug parents, and *N* = 50 control families	Explores the experiences of children with drug using parents and non-drug using parents in school settings
Poon et al., 2000	Quantitative, longitudinal	*N* = 198 boys as part of a larger longitudinal study	Examines the academic performance of children with alcoholic families
Jacob & Windle, 2000	Quantitative, comparative	*N* = 128 adults of alcoholic fathers, *N* = 138 adults of controls, *N* = 127 adults of depressed fathers	Examines academic achievement of children who had alcoholic or depressed fathers and controls
Gakhar & Jaswal, 2000	Quantitative, comparative	*N* = 60 children of alcoholics, and *N* = 60 controls	Explores academic self-concept across children of alcoholics and controls
McGrath et al., 1999	Quantitative, comparative	*N* = 221 children of alcoholics and *N* = 196 geographically matched controls	Compared the academic performance of children of alcoholics and controls
Hill et al., 1999	Quantitative, comparative	*N* = 123 children who were at high or low risk for alcoholism, taken from a large family study	Compared the academic achievement of children who were at high or low risk of alcoholism, which was determined by parental treatment of substances
Puttler et al., 1998	Quantitative, cross-sectional	*N* = 212 families drawn from a larger longitudinal study	Examined reading, spelling, arithmetic and behavior across the samples of children who had parents with alcoholism, anti-social behavior, both, and controls
Malo & Tremblay, 1997	Quantitative, comparative	*N* = 131 children in four groups across alcoholism and socioeconomic status	Investigates how parental alcoholism and socioeconomic status influences school adjustment
Hogan, 1997	Qualitative, multi-level analysis	*N* = 8 mothers, *N* = 4 fathers, *N* = 6 teachers, *N* = 10 professional works and *N* = 2 carers	Explored school outcomes and experiences of children who had drug-using parents
Vitaro et al., 1996	Quantitative, comparative	*N* = 28 sons of alcoholics, *N* = 132 sons of non-alcoholics	Compares the school achievement across sons of alcoholics and non-alcoholics
Moss et al., 1995	Quantitative, comparative	*N* = 99 sons of substance users, *N* = 78 controls	Understands the impact of parental and familial substance abuse disorders on boys' school achievement
Kolar et al., 1994	Quantitative, comparative	*N* = 70 opiate addicts in methadone maintenance treatment	Explores the school experiences and outcomes of children whose parents are on methadone maintenance treatment
Braggio et al., 1993	Quantitative, comparative	*N* = 116 patients with psychiatric illness, *N* = 53 substance users, *N* = 63 conduct disorder, and *N* = 22 controls	Examines the effect of family alcoholism history and academic achievement
Reich et al., 1988	Quantitative, comparative	*N* = 32 children of alcoholics and *N* = 22 controls	Examines the association between parental alcohol use and school achievement
Chandy et al., 1993	Quantitative, cross-sectional	*N* = 36,000 students taken from an existing adolescent health survey	Understands how self-reported parental substance use effects school enjoyment and attainment
Connolly et al., 1993	Quantitative, comparative	Data taken from existing longitudinal study. *N* = 750 and *N* = 643 teacher reports of children at age 9 and 13 respectively. *N* = 739 and *N* = 661 parent reports of children at age 9 and 13 respectively	Understands the effect of parental alcoholism on children's school behavior and grades
Sher et al., 1991	Quantitative, comparative	*N* = 253 children of alcoholics and *N* = 237 controls	Compares the class ranks and test scores for children of alcoholics and controls
Murphy et al., 1991	Quantitative, comparative	*N* = 39 children of male alcoholics and *N* = 33 controls	Examines children of alcoholics school adjustment compared to controls
Hyphantis et al., 1991	Quantitative, cross-sectional	*N* = 7,904 students taken from an existing survey	Compares academic self-concept across children with parental alcoholism vs. no alcoholism
McCarthy & Anglin, 1990	Quantitative, comparative	*N* = 756 men who were dependent on heroin	Examined the academic achievement and school drop-out of men with fathers who were frequently intoxicated
Johnson & Rolf, 1988	Quantitative, comparative	*N* = 50 children of alcoholics and *N* = 48 controls	Compares the academic performance and school enjoyment for children of alcoholics and controls
Marcus, 1986	Quantitative, comparative	*N* = 40 children of alcoholics and *N* = 40 controls	Compares the academic achievement for children of alcoholic mothers and controls
Schulsinger et al., 1986	Quantitative, comparative	*N* = 134 sons of alcoholics and *N* = 70 matched controls	Compares the school grades, school moves, school psychologist referrals and impulsive behavior across boys with alcoholic fathers and controls
Knop et al., 1985	Quantitative, comparative	*N* = 130 children of alcoholics and *N* = 70 matched controls	Examines school behavior and achievement across children of alcoholics and matched controls
Tarter et al., 1984	Quantitative, comparative	*N* = 41 adolescents, of which *N* = 16 had a father who had alcoholism, *N* = 25 matched controls	Examined a range of test scores across adolescents whose father had alcoholism and those that did not
Rydelius, 1981	Quantitative, comparative	*N* = 229 children of alcoholics and *N* = 162 control children	Examines school performance and behavior across children of alcoholics and controls
Sowder & Burt, 1980	Mixed method, quantitative descriptive and interviews	*N* = 160 parents and *N* = 160 children from treatment centers, with equal number of controls	Explores school experiences and test scores of both children whose parents are receiving treatment for substance use and for controls
Offord et al., 1978	Quantitative, comparative	*N* = 73 families	Examines school performance and attendance of 'delinquent' males whose parents were interviewed for alcoholism
Miller & Jang, 1977	Quantitative, comparative	*N* = 160 children of alcoholics and *N* = 160 controls	Compares the use of school counselling and school behavior across children of alcoholics and controls
Robins et al., 1977	Quantitative, comparative	*N* = 223 fathers, *N* = 157 children and *N* = 88 partners	Examines school attendance, behavior and repetition of school year for children of alcoholics and controls
Fine et al., 1976	Quantitative, comparative	*N* = 39 children of alcoholics and *N* = 39 matched controls	Assesses differences between children of alcoholics and controls school records
McLachlan et al., 1973	Quantitative, comparative	*N* = 54 children of alcoholics and *N* = 54 controls	Examines school test scores and academic self-concept across children of alcoholics and controls
Kammeier, 1971	Quantitative, comparative	*N* = 421 adolescents from a Catholic high school	Examines school test scores in children whose parents have an alcohol problem and those who do not
Haberman, 1966	Quantitative, comparative	*N* = 53 children from alcoholic families, *N* = 58 controls and *N* = 55 with stomach trouble	Compares the school behavior of children of alcoholics and controls
Aronson & Gilbert, 1963	Quantitative, comparative	*N* = 41 boys of alcoholics and *N* = 123 matched controls	Compares the school behavior (teacher reported) in boys of alcoholics and controls
Nylander, 1960	Quantitative, comparative	*N* = 261 children of alcoholics and *N* = 163 controls	Examines the school behavior across children of alcoholics and controls

### Attainment

Attainment was the most common outcome and the majority of studies evidenced a negative association between parental substance use and their children’s educational attainment. A number of longitudinal studies were identified, particularly in the last 10 years. Some of these studies used routine data to measure parental substance use in the form of hospital admissions, or parental alcohol problems (Berg et al., 2016; Evans et al., 2020; Gifford et al., 2015; Raitasalo et al., 2020) some of which had large population-level samples. Most studies found a negative association, for example Evans et al. (2020) found an increased risk of not attaining the expected grade at age seven years and eleven years, if the child had a parent with an alcohol admission or problem. Likewise, Raitasalo et al. (2020) found the likelihood of attainment was lower for children with parental alcohol problems, with maternal problems being a greater risk, and economic distress being a partial mediator. However, Berg et al. (2016) did not find a remaining significant negative association once models were adjusted for confounders.

Alongside this, a large number of observational studies were found. The largest study and most recent was Díaz et al. (2008) who compared 371 children of alcoholics to 147 matched controls; they found that children of alcoholics were nine times at risk of lower performance, and twice as likely to repeat a grade. Likewise, Sher et al. (1991) explored data from 253 children of alcoholics and 237 controls, and found that children of alcoholics had lower class ranks and test scores. However, a small number of observation studies did not find a significant difference. A qualitative study found that drug-using parents often were challenged by maintaining routine in the home, but teachers were mixed on their experiences of children, as some were still reaching their full potential (Hogan & Higgins, 2001). An earlier study by Hogan (1997) found that all teachers registered some concern about the attainment of children with drug-using parents, some with serious concerns which they anticipated would continue throughout the life course.

### School Behavior and Adjustment

Studies also explored school adjustment, suspensions, exclusions, truancy, and early school departure. A population-level study by Torvik et al. (2011) found that children who frequently saw their parents intoxicated were more likely to have conduct problems; this finding was mirrored in other studies for alcohol (e.g., Puttler et al., 1998; Rydelius, 1981) and drug use (Hogan & Higgins, 2001; Sowder & Burt, 1980). However, McGrath et al. (1999) did not find this, and argued that the school environment promotes positive behaviors. However, qualitative research illuminated that children of substance users often had discipline issues at school, which were defined as impulsive or angry (Hogan, 1997; Kolar et al., 1994). In addition to adjustment, suspensions and exclusions were a key feature. A large study by Jennison (2014) found that parental alcohol use was associated with early departure from school, and a threefold increased risk of suspensions; this was mirrored in other studies (e.g., Díaz et al., 2008; Miller & Jang, 1977; Pinto & Kulkarni, 2012).

### School Attendance

Some studies highlighted that the children of substance users were also at greater risk for poor school attendance (e.g., Gifford et al., 2015; McGrath et al., 1999; Sowder & Burt, 1980); this finding was found across longitudinal quantitative and cross-sectional qualitative research. Qualitative research elicited that poor attendance by some of the children had affected their educational progress, as some children had missed two months of school (Hogan, 1997; Hogan & Higgins, 2001). Likewise, Jeffreys et al. (2009) found that children experiencing parental substance use and maltreatment were more likely to be absent than children who experienced maltreatment alone.

### Academic Self-Concept

Two studies focused on child academic self-concept (Gakhar & Jaswal, 2000; Hyphantis et al., 1991) this was measured by using self-rated performance at school by children. Hyphantis et al. (1991) found that children of alcoholics were less likely to rate themselves as ‘excellent’ or ‘very good’, and more likely to rate themselves as ‘good’, ‘moderate’, or ‘bad’. Likewise, Gakhar and Jaswal (2000) found that around half of the children of alcoholics rated themselves as ‘above average’ compared to children of non-alcoholics. The authors theorize that poor academic self-concept is related to lower self-esteem, insecurity, and feelings of being inferior due to the lower quality relationships in the home when a caregiver uses substances.

### School Satisfaction and Enjoyment

Four studies explored school satisfaction and enjoyment across children whose parents used substances; the findings across studies were mixed. Torvik et al. (2011) found no association in school satisfaction; likewise, other studies found no differences in school enjoyment across children of substance-users and controls (Chandy et al., 1993; Sowder & Burt, 1980). However, Johnson and Rolf (1988) found that children of alcoholics disliked school more (28%) compared to controls (10%).

### Miscellaneous

Across the research identified, some studies investigated outcomes that were niche. For instance, lower homework completion was explored by Hogan's (1997) qualitative study. Moreover a number of studies touched on the ‘special classes’ attended by children of substance users which were related to academic progress and discipline problems (Carbonneau et al., 2017; Knop et al., 1985; Kolar et al., 1994; Malo & Tremblay, 1997). Some studies also included the use and referral to the school psychologist by children of substance users (Knop et al., 1985; Schulsinger et al., 1986; Sowder & Burt, 1980). Overall, studies suggested that the school are often aware of problems in the family home and attempt to provide early support for affected children.

## Discussion

This review was able to identify 51 empirical studies that explored the relationship between parental substance use and children’s educational outcomes. Five themes emerged in the literature which featured attainment, school behavior and adjustment, attendance, academic self-concept, school enjoyment and satisfaction, along with more miscellaneous outcomes. Attainment was the most commonly explored outcome across the studies, with children of substance using parents often attaining lower grades. Indeed, children experiencing parental substance use were more likely to have behavioral problems, lower attendance, and poorer academic self-concept. More niche findings included lower homework completion, and attendance of ‘special classes’ including referrals to the school psychologist.

While many reviews have focused on academic attainment and school behavioral problems to an extent, this review goes further to highlight how other aspects of educational outcomes are affected. For example, this review provided a more detailed account of behavioral problems in terms of conduct (Torvik et al., 2011), discipline issues (Kolar et al., 1994), attention deficit, and suspensions and exclusions (Jennison, 2014). Moreover, it documented lower attendance at school, and how qualitative work explored the impact this had (Hogan & Higgins, 2001). Attention was also given to how children perceive themselves at school, termed as academic self-concept, and how self-esteem may impact on educational outcomes. This recognition of broader educational outcomes may elude to potential mechanisms between childhood adversity and educational attainment; for example, how low attendance was described as an explanation for slower academic progress, or how challenging behavior often led to classroom removal, which decreased the time spent learning.

Moreover, this review highlighted studies which also identified how the school can be a protective factor for some children experiencing adversity. Most studies did not find a difference in school enjoyment and satisfaction between the children who had substance using parents and those who did not. Perhaps it is plausible to posit that if the school is identified as a safe environment by the child, it can be used to provide support to children experiencing substance use. Smith (1993) discusses the value of school-level interventions for children of substance users, and argues that the school can foster good teacher–pupil relationships, and provide routine when children’s family or home environment is chaotic.

From this review, the author encourages researchers to include educational outcomes when considering childhood adversity. While considerable attention has been given to the domains of mental health and wellbeing, substance use and injury, greater attention is needed on educational outcomes. Research shows educational attainment has important implications for socioeconomic position, subsequent life opportunities and health (Marmot, 2005) such as suicide (Bjorkenstam et al., 2011) or drug abuse (Gauffin et al., 2013). Given that domains of health and wellbeing can cascade to others (Masten et al., 2004) the inclusion of a broader range of outcomes would benefit how we understand and navigate childhood adversity.

While the review found considerable literature, a number of significant gaps in the field remained. First, there is a lack of research on children where parents use illicit drugs (Barnard & McKeganey, 2004) as few studies examined this, and most were dated before 2001 (Brook et al., 2010; Chandy et al., 1993; Gifford et al., 2015; Hogan, 1997; Hogan & Higgins, 2001; Jeffreys et al., 2009; Kolar et al., 1994; Moss et al., 1995; Sowder & Burt, 1980). Second, and building on the previous point, fewer studies considered parental poly-use of substances, which is a concern when Raitasalo et al. (2015) found that poly-use has a greater association with child hospital admissions. Moreover, research on parental substance use dynamics has found that behaviors can be mirrored, and heavier substance users often have a higher likelihood of poly-use (Lowthian et al., 2020). Third, studies should consider comparing estimates using different measurements of substance use (e.g., dependence vs. binge drinking) as studies largely differed; for example, some research used hospital admissions (Evans et al., 2020), dependence (Díaz et al., 2008) or quantity (Mangiavacchi & Piccoli, 2018). It is also advised that studies ensure that they adjust for key confounders, as some studies included did not do this, and this limits interpretability. Given the link between deprivation and adversity (Bellis et al., 2014) and some studies finding no association once models were adjusted (Berg et al., 2016) research must include confounders.

Lastly, very few studies considered the mechanisms, or potential mediators in the relationship between parental substance use and children’s educational outcomes. Brook et al. (2010) used a small sample to examine whether the mother–child relationship was a mediator between parental substance use and academic achievement; they found moderate evidence that substance use decreases the mother–child relationship, which was concerning, as this had a positive association with academic achievement. Given that reviews of this field often point towards household dysfunction and parenting being key explanations (Kuppens et al., 2020; McGovern et al., 2018; Park & Schepp, 2015; Velleman & Templeton, 2016) future research and interventions (e.g. Dawe et al., 2003) must evaluate the contribution of parenting and the family environment to confirm associations and pathways.

While this review has consolidated a body of research, it is limited by the non-systematic strategy used. Hence, it is strongly recommended that a systematic review is conducted, given the volume of literature found in this review that has not been identified in others. A systematic review will provide a more robust method for identifying literature, and also can evaluate the studies in terms of methodological quality. Nevertheless, this review has confirmed that most studies found a negative association between parental substance use and a broad range of children’s educational outcomes. In addition, it has identified research gaps in terms of parental drug use, poly-use and mechanisms. Following this, there are a number of research recommendations to improve our understanding, which may better inform interventions and strategies for supporting families experiencing substance use, and childhood adversity more broadly.
